# Reply to Gupta, “Reconsidering throat swab culture in melioidosis: diagnostic stewardship with clinical nuance”

**DOI:** 10.1128/jcm.00818-26

**Published:** 2026-07-20

**Authors:** Robert C. Duguid, Robert W. Baird, Jann Hennessy, Bart J. Currie, Ella M. Meumann

**Affiliations:** 1 Department of Paediatrics, Royal Darwin Hospital, Darwin, Australia; 2 Microbiology Department, Territory Pathology, Royal Darwin Hospital, Darwin, Australia; 3 Department of Infectious Diseases, Division of Medicine, Royal Darwin Hospital, Darwin, Australia; 4 Global and Tropical Health Division, Menzies School of Health Research and Charles Darwin University, Darwin, Australia; Mayo Clinic, Rochester, Minnesota, USA

## REPLY

We thank Gupta (1) for their thoughtful and well-reasoned comments regarding our study evaluating the utility of throat and rectal swab culture for melioidosis diagnosis (2).

First, we agree that the value of a diagnostic test should not be judged solely by absolute sensitivity. As the author highlights, throat swabs are minimally invasive, inexpensive, and may be of particular value in settings where access to automated blood culture systems is limited. We also agree that even modest additional diagnostic yield may be clinically important in melioidosis, given the consequences of delayed diagnosis and treatment.

Second, we agree that throat swabs remain particularly useful in selected clinical scenarios. Indeed, this was reflected in our original manuscript, where we noted their utility in patients with pneumonia who are unable to produce sputum, for example, children.

Third, the author correctly notes that the clinical manifestations of melioidosis differ between endemic regions. Upper aerodigestive tract involvement, including suppurative parotitis and cervical lymphadenitis in children, is recognized more frequently in parts of South and Southeast Asia than in northern Australia (3, 4). In such settings, the diagnostic utility of throat swabs may be higher than observed in our predominantly adult Australian cohort. We agree that diagnostic stewardship recommendations should be adapted to local epidemiology, clinical presentation, and laboratory capability.

Our study was primarily intended to investigate the utility of indiscriminate throat and rectal swab collection in all patients with suspected melioidosis within our local setting, where rectal swabs, in particular, rarely represented the sole positive diagnostic specimen. Rather than abandoning throat and rectal swab culture for melioidosis diagnosis, we support a targeted and clinically nuanced approach to their use, as suggested by Gupta.
